# Clinical features of pediatric mucormycosis: role of metagenomic next generation sequencing in diagnosis

**DOI:** 10.3389/fcimb.2024.1368165

**Published:** 2024-06-10

**Authors:** Yu Zhang, Erhu Wei, Jiechao Niu, Kunli Yan, Mengjiao Zhang, Wenhua Yuan, Xiao Fang, Peisheng Jia

**Affiliations:** Department of Pediatrics, The First Affiliated Hospital of Zhengzhou University, Zhengzhou, Henan, China

**Keywords:** mucormycosis, clinical features, MNGs, diagnosis, children

## Abstract

**Background:**

Mucormycosis is an uncommon invasive fungal infection that has a high mortality rate in patients with severe underlying diseases, which leads to immunosuppression. Due to its rarity, determining the incidence and optimal treatment methods for mucormycosis in children is challenging. Metagenomic next-generation sequencing (mNGS) is a rapid, precise and sensitive method for pathogen detection, which helps in the early diagnosis and intervention of mucormycosis in children. In order to increase pediatricians’ understanding of this disease, we conducted a study on the clinical features of mucormycosis in children and assessed the role of mNGS in its diagnosis.

**Methods:**

We retrospectively summarized the clinical data of 14 children with mucormycosis treated at the First Affiliated Hospital of Zhengzhou University from January 2020 to September 2023.

**Results:**

Of the 14 cases, 11 case of mucormycosis were classified as probable, and 3 cases were proven as mucormycosis. Most children (85.71%) had high-risk factors for mucormycosis. All 14 children had lung involvement, with 5 cases of extrapulmonary dissemination. Among the 14 cases, 4 cases underwent histopathological examination of mediastinum, lung tissue or kidney tissue, in which fungal pathogens were identified in 3 patients. Fungal hyphae was identified in 3 cases of mucormycosis, but only 1 case yielded a positive culture result. All patients underwent mNGS testing with samples from blood (8/14), bronchoalveolar lavage fluid (6/14), and tissue (1/14). mNGS detected fungi in all cases: 7 cases had *Rhizomucor pusillus*, 4 cases had *Rhizopus oryzae*, 3 cases had *Rhizopus microsporus*, 1 case had *Lichtheimia ramosa*, and 1 case had *Rhizomucor miehei*. Coinfections were found with *Aspergillus* in 3 cases, bacteria in 3 cases, and viruses in 5 cases.

**Conclusion:**

Children with mucormycosis commonly exhibit non-specific symptoms like fever and cough during the initial stages. Early diagnosis based on clinical symptoms and imaging is crucial in children suspected of having mucormycosis. mNGS, as a supplementary diagnostic method, offers greater sensitivity and shorter detection time compared to traditional mucormycosis culture or histopathological testing. Additionally, mNGS enables simultaneous detection of bacteria and viruses, facilitating timely and appropriate administration of antibiotics and thereby enhancing patient outcomes.

## 1 Introduction

Mucormycosis, a severe fungal infection caused by Mucorales fungi, aggressively invades human blood, organs, and tissues. It poses a significant threat to children with suppressed immune function post-transplantation, with a high mortality rate (Cornely et al., 2019; Hassan and Voigt, 2019). Recent epidemiologic research reveals that mucormycosis is the third most prevalent invasive fungal disease among children trailing behind aspergillosis and candidiasis (Francis et al., 2018). There has been a notable increase in mucormycosis cases over recent years (Bitar et al., 2014), with rates in Asia ranging from 1 to 12.3 per million (Hassan and Voigt, 2019).The condition predominantly impacts individuals with diabetes or compromised immunity, including those with hematologic malignancies, transplant recipients, and patients who have undergone surgery, experienced burns, or suffered trauma (Feng and Sun, 2018).

Underlying disease is a major influence on the development of pediatric mucormycosis. Among children with trichinosis, 46% had a history of hematological malignant disease, 46% had a history of neutropenic disease, 15.9% had been treated with hematopoietic stem cell transplantation (HSCT), 4.8% had been treated with solid organ transplantation, and 4.8% had a history of diabetes (Pana et al., 2016; Skiada et al., 2018). Pediatric mucormycosis, especially in patients presenting with mucormycosis after HSCT, are usually immune-compromised and have a rapid progression of the disease. Therefore, a comprehensive treatment model with rapid etiological diagnosis, correction of susceptibility factors, early surgical debridement and systemic antifungal therapy is essential to improve prognosis and survival (Olivier-Gougenheim et al., 2021).

Identifying mucormycosis early is a challenge due to its non-specific symptoms and signs. Current diagnoses primarily rely on imaging, histopathology, and mycological culture. The varied pathogenic characteristics of mucormycosis, similar to other invasive fungal infections, make diagnosis difficult (Chamilos et al., 2005; Lass-Florl et al., 2007). Histopathology or culture is considered the “gold standard” for diagnosis, but due to sampling difficulties and limitations of culture methods, only about 50% of cases yield positive results (Roden et al., 2005; Walsh et al., 2012). Grocott’s Methenamine Silver (GMS) is preferred in mucormycosis and can be shown in tissue specimen sections as broad, irregular, unseparated or minimally segregated or right-angled branched hyphae (Goldberg et al., 2015). In contrast, performing definitive species identification requires molecular methods (mainly through sequencing of internal transcribed spacer regions), or matrix-assisted laser desorption ionization-time of flight mass spectrometry (Danion et al., 2023). Other methods like the (1–3)-β-D-glucan assay (G test) and polymerase chain reaction (PCR) have limitations in accurately diagnosing mucormycosis (Bellanger et al., 2011). Currently, there are no serological tests or serum biomarkers available for early diagnosis, thus necessitating the need for new methods (Stone et al., 2021).

Metagenomic next-generation sequencing (mNGS), a modern molecular biology technique, has emerged as a promising tool. It is capable of identifying over 15,000 pathogen species with known genomic sequences (Gu et al., 2019). mNGS offers high sensitivity, short detection times, and the ability to diagnose rare pathogen infections, significantly enhancing pathogen detection rates in clinical environments (Grumaz et al., 2016; Zheng et al., 2021).

This study conducts a retrospective analysis of the clinical features, treatment approaches, and outcomes of 14 pediatric mucormycosis cases. It aims to assess the characteristics and treatment efficacy and explore the potential of mNGS in early diagnosis of mucormycosis in children.

## 2 Methods

### 2.1 Study design and participants

This retrospective study included 14 children with mucormycosis hospitalized at the Children’s Hospital of the First Affiliated Hospital of Zhengzhou University from January 2020 to September 2023. The inclusion criteria were as follows: (1) Children proven or probable to have mucormycosis according to the definitions of invasive fungal diseases by the European Organization for Research and Treatment of Cancer/Mycoses Study Group of the National Institute of Allergy and Infectious Diseases (Donnelly et al., 2020). “Proven” cases required positive results from mucormycosis culture and/or histopathological examination. For “probable” mucormycosis, a joint diagnosis by imaging experts and clinical doctors of the hospital was needed; (2) Completion of mNGS testing. Patients who meet the following criteria were excluded: (1) age ≥18 years, (2) incomplete medical records. Furthermore, data on patients’ baseline, clinical features, laboratory and imaging information, diagnosis, treatments, and outcomes were collected.

### 2.2 mNGS protocol

Clinical samples, such as blood, bronchoalveolar lavage fluid, or lung tissue, were collected using aseptic techniques. Nucleic acids were extracted using the TIANamp Micro DNA Kit (DP316) from Tiangen Biotech Co., Ltd. (Beijing, China). A total of 100 ng of the extracted DNA underwent fragmentation, end repair, library construction, and sequencing. Quality assessment was performed using the Agilent 2100 system. Sequencing was conducted on the BGISEQ-100 platform at the Beijing Genomics Institute (Wuhan, China). After mapping human sequences to the human reference genome (hg19) using the Burrows–Wheeler Alignment, non-human sequences were analyzed. Reads with low quality or shorter than 35 base pairs were discarded. The remaining sequences were compared against four microbial genome databases, including bacteria, viruses, fungi, and parasites. Comprehensive data analysis was conducted on the aligned sequences. Potential pathogens were identified, and their data were listed, including the number of precisely mapped reads, coverage, and depth. The final clinical diagnosis was determined by integrating these findings with clinical symptoms and other laboratory test results.

## 3 Results

### 3.1 Baseline characteristics

This study initially included 20 children suspected of having mucormycosis. Six children were excluded due to the absence of mNGS testing and incomplete data, resulting in 14 children being selected for the study. 8 cases (57.14%) were male among these children. The median age of the participants was 13.00 years, with a range of 7.00 to 14.00 years. **Table 1** lists the clinical characteristics of these patients. Among them, 11 cases (78.57%) had hematologic malignancies, 1 case (7.14%) had a mediastinal tumor, 1 case (7.14%) had diabetes, and 1 case (7.14%) had no underlying diseases.

**Table 1:** Demographic and clinical characteristics of mucormycosis patients.

	Case 1	Case 2	Case 3	Case 4	Case 5	Case 6	Case 7	Case 8	Case 9	Case 10	Case 11	Case 12	Case 13	Case 14
Gender	M	M	M	M	M	F	M	F	M	F	F	F	F	M
Age, y	15	13	14	10	8	14	4	2	14	14	5	7	14	13
Underlying disease	Diabetes	MT	No	ALL	ALL	ALL	ALL	ALL	AML	MS	ALL	ALL	T-ALL/LBL	AA
Symptoms														
Fever	Yes	Yes	Yes	Yes	Yes	Yes	Yes	Yes	Yes	Yes	Yes	Yes	Yes	Yes
Cough	No	Yes	Yes	No	Yes	No	No	Yes	Yes	Yes	Yes	No	No	No
Sputum	No	No	No	No	No	No	No	Yes	No	No	No	No	No	No
Chest pain	No	No	No	No	No	No	No	No	No	Yes	No	No	No	No
Abdominal pain	No	No	No	No	No	No	No	No	No	No	No	No	No	Yes
Neutropenia	No	No	Yes	Yes	Yes	No	Yes	Yes	Yes	Yes	Yes	No	No	Yes
Coma	No	No	No	No	No	No	No	No	No	No	No	Yes	Yes	Yes
Convulsions	No	No	No	No	No	No	No	No	No	No	No	No	Yes	No
Mycological evidence														
G	N	N	NA	N	N	N	N	N	N	N	N	N	N	N
GM	N	N	N	N	N	N	N	N	N	N	N	N	N	N
BALF Culture	P	N	N	N	N	N	N	N	N	N	N	N	N	N
BALF IF	P	NA	N	NA	NA	NA	P	P	P	NA	N	NA	N	NA
Histopathologic	NA	P (mediastinum)	N (lung)	NA	NA	P (lung, kidney)	NA	NA	NA	NA	P (lung)	NA	NA	NA
BALF mNGS (Sequences Number)	*Rhizopus oryzae* (7); *Aspergillus fumigatus* (105)	NA	*Rhizopus oryzae* (88)	NA	NA	*Lichtheimia ramose* (1); *Cytomegalovirus* (1)	NA	NA	*Rhizopus oryzae* (217); *Human betaherpesvirus* 1(6342); *Human betaherpesvirus* 4(3)	NA	*Rhizopus microspores* (4); *Haemophilus influenzae* (68)	NA	*Rhizopus microspores* (1106); *Enterococcus faecium* (99); *Human betaherpesvirus* 5(234)	NA
Blood mNGS(Sequences Number)	NA	NA	NA	*Rhizomucor pusillus* (463); *Aspergillus fumigatus* (1)	*Rhizomucor pusillus* (1358); *Human betaherpesvirus 1* (116)	NA	*Rhizomucor pusillus* (16672); *Aspergillus terreus* (8)	*Rhizomucor pusillus* (1410)	*Rhizopus oryzae* (1972); *Human betaherpesvirus* 1(334); *Human betaherpesvirus 7*(2)	*Rhizomucor pusillus* (1425); *Human betaherpesvirus* 7 (1)	NA	*Rhizomucor miehei*(4); *Rhizomucor pusillus* (6)	NA	*Rhizomucor pusillus* (3116); *Mycobacterium* (2)
Tissue mNGS(Sequences Number)	NA	*Rhizopus microspores* (30);	NA	NA	NA	NA	NA	NA	NA	NA	NA	NA	NA	NA
Clinical forms of mucormycosis	Pulmonary	Pulmonary	Pulmonary	Pulmonary	Pulmonary	Disseminated	Disseminated	Pulmonary	Pulmonary	Disseminated	Pulmonary	Disseminated	Pulmonary	Disseminated
Classification of diagnosis	Probable	Proven	Probable	Probable	Probable	Proven	Probable	Probable	Probable	Probable	Proven	Probable	Probable	Probable
Treatment	L-AmB and posaconazole	L-AmB	isavuconazol	L-AmB and posaconazole	L-AmB and posaconazole	posaconazole	L-AmB	L-AmB and posaconazole	L-AmB and posaconazole	L-AmB and posaconazole	L-AmB and posaconazole	L-AmB and posaconazole	L-AmB and posaconazole	L-AmB and posaconazole
Surgery	No	No	No	No	No	Lobectomy	Vacuum Sealing Drainage	No	No	No	Lobectomy	No	No	No
Outcome	Death	Death	Alive	Alive	Death	Alive	Alive	Alive	Death	Death	Alive	Alive	Death	Alive

M, male; F, female; MT, mediastinal tumor; ALL, acute lymphocytic leukemia; AML, acute myeloid leukemia; MS, Myeloid sarcoma; T-ALL/LBL, T-cell acute lymphoblastic leukemia/lymphoma; AA, Aplastic anemia; IF, immunofluorescence; G, 1,3 beta-D glucan detection test; GM, galactomannan test; mNGS, Metagenomic next-generation sequencing; N, negative; P, positive; NA, not applied; L-AmB, liposomal amphotericin B.

**Figure 1:**
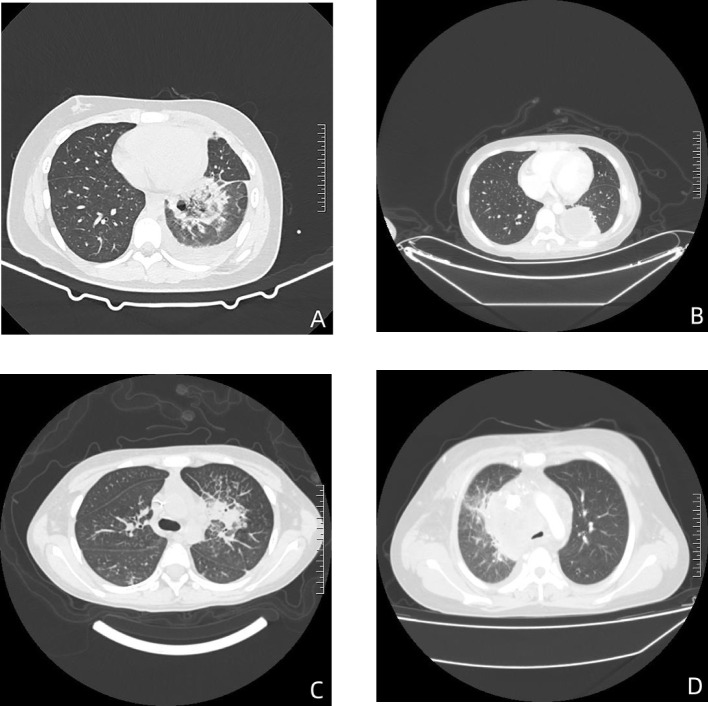
The chest CT findings of children with mucormycosis. **(A)** In the lower lobe of the left lung, there’s a mass-like area of high density with an internal cavity. This suggests a localized infection with tissue destruction leading to cavity formation. **(B)** In the lower left lung, there’s an encapsulated fluid density shadow accompanied by some consolidation. This could indicate an abscess or a localized collection of fluid, possibly due to infection. **(C)** There’s evidence of obstructive pneumonia in the upper lobe of the left lung, with partial narrowing of the upper lobe bronchus and multiple patchy areas of high density observed distally. This is indicative of infection causing obstruction in the airways. **(D)** In the mediastinum, there’s an unclearly demarcated mass-like soft tissue density shadow. Additionally, there’s narrowing and truncation of the right upper lobe bronchus and multiple solid nodules in both lungs. This could represent the spread of infection or inflammatory response in the mediastinum and lungs.

All children presented with fever, and other symptoms included cough (7 cases, 50.00%), coma (3 cases, 21.43%), convulsions (1 case, 7.14%) and chest pain (1 case, 7.14%). At the time of diagnosis, 9 children (64.29%) had neutropenia. The main chest CT findings included (**Figure 1**): pleural effusion in 7 cases (50.00%), consolidation in 5 cases (35.71%), nodules in 4 cases (28.57%), cavities in 2 cases (14.29%), and abscess in 1 case (7.14%).

Based on the bronchoalveolar lavage (BAL) diagnosis, 13 cases (78.57%) of mucormycosis were classified as probable, and 3 cases (21.43%) were proven as mucormycosis. Of the 9 cases (64.29%) with invasive pulmonary mucormycosis, 4 cases had acute lymphocytic leukemia (ALL), 1 (7.14%) had concurrent acute myeloid leukemia (AML), 1 case (7.14%) had T-cell acute lymphoblastic leukemia/lymphoma (T-ALL/LBL), 1 case (7.14%) had diabetes, and 1 case (7.14%) had a mediastinal tumor.

### 3.2 Culture, pathology, and NGS findings

**Figure 2:**
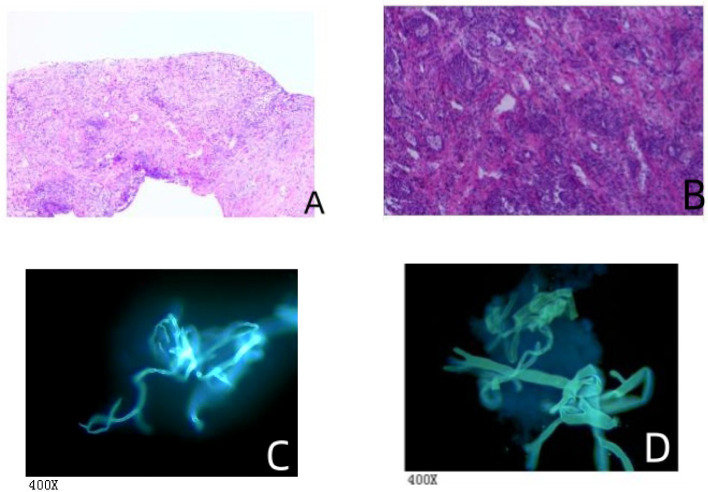
The histopathological and fungal immunofluorescence microscopy findings. **(A)** Mediastinal biopsy showed chronic inflammation with necrosis in fibrous tissue and the presence of a few fungal hyphae, suggestive of mucormycosis. Special staining results were: Acid-fast bacilli (AFB) negative, Periodic Acid-Schiff (PAS) positive, and Gomori methenamine silver (GMS) positive. This indicates the presence of mucor species, as evidenced by the PAS and GMS positivity. **(B)** Lung interstitial fibrous tissue showed hyperplasia with infiltration of acute and chronic inflammatory cells. There were tissue cells aggregating in the alveolar spaces, extensive infarction with inflammatory necrosis, and small focal granulomas, consistent with an inflammatory lesion. Fungal hyphae and spores were observed in the necrotic tissue, indicative of fungal infection. Special staining results were also AFB negative, PAS positive, and GMS positive, confirming the presence of fungal elements. **(C)** Bronchoalveolar lavage fluid (BALF) examination revealed non-septate hyphae branching at 90° angles, suggestive of mucormycosis-like fungal filaments. **(D)** Another BALF sample similarly showed non-septate hyphae with right-angle branching, indicative of mucormycosis-like fungal filaments.

All cases underwent cultures of sputum, or bronchoalveolar lavage fluid, but only one case yielded a positive culture. Among the 14 cases, 4 children (28.57%) underwent histopathological examination, in which fungal pathogens were identified in 3 patients. Fungal hyphae was identified in 2 cases of pulmonary mucormycosis and 1 case of disseminated mucormycosis (**Figure 2**). Fungal hyphae was also found in 4 cases (28.57%) using immunofluorescence microscopy (**Figure 2**). And all cases tested negative in GM and G tests. All 14 cases underwent cultures from bronchoalveolar lavage fluid or sputum, but only 1 case (7.14%) yielded a positive culture result. The mNGS test is more sensitive than conventional diagnostic methods (*P*<0.001), in **Table 2**.

**Table 2:** Comparison of mNGS and conventional detection of mucormycosis.

	Culture	IF	Histopathologic	mNGS	*F*	*P*
Negative,n	13	3	1	0	27.898	<0.001
Positive,n	1	4	3	14		
total,n	14	7	4	14		

IF, immunofluorescence; mNGS, Metagenomic next-generation sequencing.

mNGS was performed on all cases, using samples primarily from blood and bronchoalveolar lavage fluid, but also from tissue at the site of infection (**Table 2**). Among the 14 patients, 7 cases (50.00%) were positive for *Rhizomucor pusillus*, 4 cases (28.57%) for *Rhizopus oryzae*, 3 cases (21.43%) for *Rhizopus microsporus*, 1 case (7.14%) for *Lichtheimia ramosa*, and 1 case (7.14%) for *Rhizomucor miehei*. Additionally, *Aspergillus* was detected in 3 cases (21.43%), bacteria in 3 cases (21.43%), and viruses in 5 cases (35.71%), all of which were cytomegalovirus.

### 3.3 Treatment and prognosis

Prior to their diagnosis of mucormycosis, 40% of the patients had undergone prophylactic antibiotic treatment. After being diagnosed with mucormycosis, all patients received antifungal therapy. Specifically, 10 patients (71.43%) were treated with a combination of Liposomal Amphotericin B (L-AmB) and posaconazole, 2 patients (14.29%) were treated with L-AmB monotherapy, 1 patient (7.14%) received isavuconazole monotherapy, and 1 patient (7.14%) was treated with posaconazole. In addition, 12 patients (85.71%) received broad-spectrum antibiotic treatment.

Surgical intervention was undertaken in 3 patients (21.43%), with two children undergoing lobectomy of the lung. Due to disease progression, 8 patients (57.14%) were transferred to the Intensive Care Unit. 6 cases (42.86%) died, while 8 cases (57.14%) showed improvement within 3 months of hospital discharge.

## 4 Discussion

This retrospective analysis focused on the clinical characteristics and mNGS diagnostic effectiveness in 14 mucormycosis children. The study found that 12 of these patients had known risk factors for mucormycosis, with 11 cases involving hematologic malignancies and 1 case with no underlying disease. One notable case in this study (Case 3) involved a previously healthy child with no history of underlying disease, immunosuppressive drug use, trauma, or other high-risk factors typically associated with mucormycosis. This child developed lung infection following non-specific symptoms such as fever, cough, dizziness, and headache, with the etiology identified as *Rhizopus oryzae* infection. Therefore, mucormycosis should also be considered in otherwise healthy children.

Mucormycosis can target various body parts, including rhino-orbital-cerebral mucormycosis (ROCM), pulmonary mucormycosis (PM), skin/soft tissue infections (SSTI), gastrointestinal or renal infections (GI), disseminated mucormycosis, and infections in atypical locations. Disseminated infection was defined as infection at ≥ 2 non-contiguous sites (Roden et al., 2005). Underlying disease is a major influence on the development of mucormycosis in children, and the clinical type of mucormycosis can vary from one underlying disease to another (Pana et al., 2016; Skiada et al., 2018). Pulmonary mucormycosis is notably prevalent in hematologic malignancies and neutropenia patients (Jeong et al., 2019).The mortality rate for mucormycosis ranges from 40% to 80%, influenced by the patient’s underlying conditions and infection site (Cornely et al., 2019). The risk of death is higher in individuals with major risk factors compared to those with other diseases (Kennedy et al., 2016; Jestin et al., 2021). Epidemiological studies on pediatric mucormycosis are limited, with mortality rates of 26.5%-33.3% reported in children with hematologic malignancies (Pana et al., 2016; Ziaka et al., 2022). In our study, 6 children (42.86%) died within 3 months of discharge, all having underlying diseases: 4 with hematologic malignancies, 1 with a mediastinal tumor, and 1 with diabetes.

Pulmonary mucormycosis typically presents with non-specific symptoms like fever, cough, breathing difficulties, and chest pain (Danion et al., 2015). The infection often affects the lung parenchyma and may spread to the chest wall, pulmonary artery, aorta or pericardium, and infiltration into the pulmonary artery can cause hemoptysis (Steinbrink and Miceli, 2021). In this study, all 14 children had lung involvement, predominantly presenting with fever, cough, and chest pain, with 9 patients experiencing neutropenia. Chest CT scans revealed consolidation as the most frequent presentation of PM, alongside mass lesions, nodules, and cavities. The main chest CT findings in the children with mucormycosis in this study were pleural effusion and consolidation, not limited to patients with neutropenia.

Overall, for patients with suspected pulmonary mucormycosis in hematologic malignancies, clinical symptoms and pulmonary imaging may not present typically. However, clinicians should be vigilant for signs of consolidation in lung CT scans or vascular blockages in CT pulmonary angiography (Busca et al., 2012).

Early diagnosis and treatment can help reduce mortality in patients with mucormycosis (Sipsas et al., 2018). However, the diagnosis relies on histopathology and culture. GMS staining is preferred, as mucormycosis can appear in tissue samples as broad, irregular, non-septate, or minimally sparsely septate hyphae, often branching at right angles (Frater et al., 2001; Goldberg et al., 2015). Even if histopathological examination shows characteristic changes of mucormycosis, tissue cultures often turn out negative, and blood cultures are usually not positive (Hammer et al., 2018). Serological tests like the GM-test and G-test), commonly used for detecting fungal infections, are often negative in mucormycosis patients (Pyrgos et al., 2008; Lass-Florl et al., 2021).

In our study, all cases underwent cultures of sputum, or bronchoalveolar lavage fluid, but only one case yielded a positive culture. Pathogens were found in the histopathological examination of only three cases, and all cases tested negative in GM and G tests. In this study, mNGS was used to detect pathogens in children’s peripheral blood, bronchoalveolar lavage fluid, and tissue from the infection site, and all children were found to be infected with mucormycosis. In a retrospective study of mNGS for the detection of pathogens in lung infections, it was found that BALF mNGS greatly improved the accuracy and detection of pathogens in patients with lung infections (Wu et al., 2022). Therefore, the value of BALF mNGS should be focused on children suspected of having a pulmonary mucormycosis. In this study it was found that the mNGS test is more sensitive than Conventional microbiological tests (*P*<0.001). In a study that included 310 patients with suspected pulmonary invasive fungal infections, it was found that compared with Conventional microbiological tests, mNGS was superior in its diagnostic performance (*AUC*=0.967) (Wang et al., 2022).mNGS has proven the presence of mucormycosis at the molecular biology level, providing a basis for initiating early antifungal treatment against mucormycosis. Before the application of mNGS, conventional diagnostic methods had low success rates in identifying mucormycosis, and treatment was more reliant on clinical experience.

While most mNGS samples in our study came from children’s peripheral blood, it doesn’t imply that the blood is the infection site. In a localized infection, mucor DNA fragments can access the bloodstream easily (Li et al., 2021). Simultaneously, mNGS is capable of detecting bacteria, fungi and viruses. In a study that included 13 children, mNGS was found to detect both fungi and bacteria in 53.85% of samples, and both fungi and viruses in 38.46% of samples (Zhang et al., 2022). In our study, 35.71% of the children were found to have viral infections, 21.43% had concurrent Aspergillus infections, and 21.43% had bacterial infections. Hence, the mNGS results should be interpreted alongside clinical symptoms and imaging to pinpoint infection presence and location, particularly when indicating multiple pathogen infections. Based on the mNGS results, the appropriate and timely use of antibiotics and antiviral treatments for patients with mixed infections better controlled the symptoms. Of course, further research is needed to understand the clinical significance of low pathogen sequence numbers in children detected by mNGS.

Prompt administration of effective antifungal treatments and the surgical excision of necrotic tissue is crucial in preventing further damage to tissues and organs in mucormycosis patients, potentially decreasing long-term complications and improving survival chances (Jeong et al., 2015; Sipsas et al., 2018). In our study, 40% of children received prophylactic antifungal treatment before the onset of mucormycosis. All cases with potential breakthrough infections had underlying hematologic malignancies, which is close to the incidence of breakthrough invasive mucormycosis reported by Skiada et al. in HSCT patients despite having posaconazole prophylaxis (Skiada et al., 2020). 85.71% (12/14) of the children in our study were initially treated with L-AmB, with 10 patients undergoing a combination treatment of L-AmB and posaconazole, and one patient (Case 3) receiving initial treatment with isavuconazole. In instances of severe, refractory or progressing mucormycosis, combining L-AmB with posaconazole has shown beneficial results (Cornely et al., 2014, 2019). However, a separate study indicated that initiating combination therapy with L-AmB and posaconazole was not able significantly to reduce mortality rates in a cohort of patients with confirmed hematologic malignancies (Kyvernitakis et al., 2016). Therefore, further research is needed to assess the potential benefits of L-AmB or isavuconazole as monotherapy or in combination treatments, based on patient outcomes and drug tolerance.

This study’s limitations must be acknowledged. First, as a single-center retrospective analysis, it inherently carries certain biases. Second, since some mNGS samples were derived from patients’ bronchoalveolar lavage fluid, it is challenging to determine whether the microbes reported by mNGS are clinically significant pathogens or merely colonizing organisms. The data generated by mNGS need to be parsed by sophisticated bioinformatics tools, and the results are limited by the completeness of the databases and current scientific knowledge. Therefore, it is important to give due consideration to the clinical setting when interpreting mNGS data. Standardized operational and parsing processes for mNGS are not yet fully established, which may affect the accuracy and reproducibility of the results. Finally, the results lack a consensus, and the diagnosis of mucormycosis according to guidelines is probable, as further histopathology or culture confirmation could not be pursued due to patient condition limitations.

In conclusion, mucormycosis in children is rare but carries a high mortality risk. Early in the disease course, it initially manifests with non-specific symptoms like fever and cough. Children suspected of mucormycosis based on clinical presentation and imaging results should be diagnosed early. Compared to traditional mucormycosis culture or histopathological testing, mNGS offers higher sensitivity and a shorter detection period, making it a supplementary method for early diagnosis. mNGS can also aid in detecting mixed infections and informing timely antimicrobial therapy, thus improving patient outcomes. Therefore, the mNGS testing method holds significant value in the early diagnosis of mucormycosis in children.

## Data availability statement

The original contributions presented in the study are included in the article/supplementary materials, further inquiries can be directed to the corresponding author/s.

## Ethics statement

The studies involving humans were approved by the ethics committee of the First Affiliated Hospital of Zhengzhou University. The studies were conducted in accordance with the local legislation and institutional requirements. The ethics committee/institutional review board waived the requirement of written informed consent for participation from the participants or the participants’ legal guardians/next of kin because this was a retrospective study and written informed consent was exempted.

## Author contributions

YZ: Data curation, Writing – original draft, Writing – review & editing. EW: Funding acquisition, Methodology, Writing – review & editing. NJ: Data curation, Formal analysis, Writing – review & editing. KY: Methodology, Writing – review & editing. MZ: Methodology, Writing – review & editing. WY: Methodology, Writing – review & editing. XF: Methodology, Writing – review & editing. PJ: Methodology, Supervision, Writing – review & editing.
